# Thyroid-Stimulating Hormone Regulates the Glucose Metabolism in Hepatocytes via Toll-Like Receptor 4/Tollip Pathway

**DOI:** 10.1155/ije/5528193

**Published:** 2025-10-15

**Authors:** Suqing Bao, Fengbo Li, Lijun Duan, Xia Jiang

**Affiliations:** ^1^Department of Endocrinology, Tianjin First Central Hospital, Tianjin 300192, China; ^2^Department of Orthopedics, Tianjin Hospital, No. 406, Jiefang South Road, Tianjin 300211, China

**Keywords:** glucose metabolism, hepatocyte, thyroid-stimulating hormone, Toll-like receptor 4, Tollip

## Abstract

Metabolic disorders are closely associated with thyroid dysfunction and the activity of thyroid-stimulating hormone (TSH). Previously, we found that subclinical hypothyroidism aggravates Toll-like receptor 4 (TLR4) signaling and interferes with glucose metabolism in rat liver tissue. Here, we explored the underlying mechanisms by which TSH affected TLR4 and glucose metabolism on hepatocytes in vitro. Hepatocytes were stimulated with TSH (0, 5, 10, and 20 mIU/mL) for 12 h and mRNA level of its receptor, thyroid-stimulating hormone receptor (TSHR), was increased. In contrast, glucose metabolism was blocked. After blocking TSHR, glucose metabolism in hepatocytes was rescued. Additionally, TSH treatment also activated TLRs signaling, and the expression of TLR4 and its downstream partners all decreased after TSHR was silenced, which indicated that TSH promotes TLR4 signaling through a TSHR-dependent mechanism. For the exploitation of the underlying relationship between TLR4 and glucose metabolism, siRNA was utilized to silence TLR4. After silencing TLR4, glucose metabolism was significantly rescued, which indicated that TLR4 was involved in the TSH-mediated downregulation of glucose metabolism in hepatocytes. Furthermore, as for the inhibitor of TLRs, Tollip was also measured. Under TSH treatment, the expression level of Tollip decreases. After silencing Tollip, TLR4 and its partners significantly increased and glucose metabolism was reduced. Our study indicated that TSH/TSHR regulated hepatocellular glucose metabolism via the TLR4/Tollip pathway.

## 1. Introduction

Thyroid disease and diabetes are endocrine disorders associated with metabolic syndrome [1–3]. Hypothyroidism is more prevalent in the diabetic population than in healthy individuals [4], accompanying with lipotoxicity and abnormal secretion of adipokines and inflammatory cytokines, thus leading to abnormal lipid and glucose metabolism [5, 6]. Subclinical hypothyroidism (SCH) predisposes a patient to metabolic syndrome [7–9] and resulted in mildly elevated levels of thyroid-stimulating hormone (TSH), while elevated TSH is found in obese patients [10]. TSH suppresses appetite and affects thermogenesis, adipogenesis, and lipolysis/lipogenesis balance, which indicated that TSH played a pivotal role in regulating energy metabolism [6]. TSH and insulin levels were correlated with insulin resistance, eventually influencing the glucose metabolism [9, 11]. Therefore, it is critical to understand the underlying mechanism by which thyroid dysfunction contributes to dysfunction of glucose metabolism.

Disrupted glucose metabolism is commonly associated with chronic low-grade inflammation in obesity [12, 13]. Toll-like receptor 4 (TLR4) is a pattern recognition receptor of the immune system and a key connection between inflammation and metabolism [14]. Numerous studies have shown that the signaling pathway of TLR4 and nuclear factor κB (NF-κB) is involved in glucose metabolism [15, 16]. Independently, TSH can activate the TLR4/NF-κB inflammatory signaling pathway in animal models of arteriosclerosis [17]. TLR4 expression is increased in peripheral blood mononuclear cells (PBMCs) of a rat model of SCH, which further confirmed that TLR signaling activated inflammation in SCH [18]. However, whether the TLR signaling pathway is involved in human hepatic glucose metabolism in SCH is still unclear.

Tollip is a selective autophagy receptor and an important endogenous inhibitor of TLR4 signaling [19–21]. Tollip and IRAK form a complex that blocks TLR signaling. Under certain conditions, Tollip is targeted for degradation via ubiquitination, which releases interleukin-1 receptor-associated kinase (IRAK) for phosphorylation, thereby activating TLR signals [20, 21]. Liver plays an important role in systemic glucose and lipid metabolism as the peripheral target organ of insulin [22, 23]. Our previous study in SCH rats demonstrated that as the level of Tollip decreased, IRAK phosphorylation increased, TLR4/NF-κB signaling was activated, and expression of inflammatory cytokines like TNF-α and IL-6 increased in the liver, while key insulin signaling proteins such as glucose transporter 2 (GLUT2) and phosphorylation of insulin receptor substrate (IRS) increased [24]. Further studies are needed on the underlying mechanism of how elevated TSH regulates these factors.

Here, we perform overexpression and silencing experiments in a hepatocyte culture model to assess the effect of TSH on TLR4 activity and glucose metabolism. The study aims to provide new insights into the links among molecular events in TSH, glucose metabolism, and immune-related inflammation.

## 2. Materials and Methods

### 2.1. Cell Culture and Construction of Cell Model

Human hepatocyte cell line, WRL-68, was obtained from the American Type Culture Collection (ATCC® CL-48™, USA) and cultured in MEM (Gibco, USA) supplemented with 5% fetal bovine serum (HyClone, USA) and 100 U/mL penicillin (Sigma, USA) in a humidified incubator (37°C, 5% CO_2_).

### 2.2. Determining TSH Concentration

Human TSH was reconstituted in sterile water to reach the final concentration. WRL 68 cells were stimulated with TSH (0, 5, 10, or 20 mIU/mL) for 12 h and collected for subsequent mRNA extraction and further analysis.

#### 2.2.1. Cellular ^3^H-2-Deoxy-D-glucose Uptake Measurements After Insulin Stimulation

WRL 68 cells were seeded at a density of 1 × 10^5^ cells per well in 24-well plates and incubated for 2 h at 37°C in serum-free medium. Following this, we treated the cells with TSH (10 mIU/mL) and incubated them for 12 h. Then, the cells were washed twice with Krebs–Ringer phosphate (KRP) buffer (pH 7.4) and subsequently incubated in KRP buffer containing insulin (100 nM) for 30 min. Next, we added ^3^H-2-deoxy-D-glucose (2 μCi/mL, Beijing Yuan Zi Gao Ke Corporation, China) to the cells and incubated them for an additional 10 min at 37°C. The reaction was stopped by three times of washing with ice-cold PBS. Cells were then solubilized in 0.5 M NaOH (0.4 mL/well) for 2 h. The solubilized samples were subjected to scintillation counting to measure ^3^H radioactivity, expressed as disintegrations per minute (DPM). Lastly, the glucose uptake was estimated by calculating DPM per milligram of protein.

### 2.3. siRNA Knockdown of TSHR, TLR4, and Tollip

Primers for inhibition of TSHR and Tollip expression via siRNA: SiTSHR-F: CCACGGAUGUGUUCUUUAUUU, SiTSHR-R: AUAAAGAACACAUCCGUGGUU; si Tollip-F: CUGCCAUCAACUCCUUACUUU, si Tollip-R: AGUAAGGA GUUGAUGGCAGUU; SiTLR4-F: CGAGCUGGUAAAGAAUUUAUU, SiTLR4-R: UAAAUUCUUU ACCAGCUCGUU. The culture medium of WRL68 cells was changed to opti-MEM serum-free medium (Gibco, USA) after a 2× wash with PBS. Cells were transfected with a Lipofectamine TM2000/siRNA complex according to manufacturer protocols and incubated for 48 h, after which the cells were washed to remove the transfection complex.

### 2.4. Process of Tollip and TLR4 Overexpression on WRL68 Cells

WRL68 cells were seeded in 12-well plates and transfected with a Tollip or TLR4 overexpression plasmid (RiboBio, China) using Lipofectamine 2000™ according to manufacturer instructions. Cells were incubated for 16–24 h at 37°C.

### 2.5. Detection of IL-6 and TNF-α

Cell supernatants were collected following treatment. The IL-6 and TNF-α concentrations were determined by an ELISA kit (Cusabio, Wuhan, China) per the manufacturer protocol. The intraassay and interassay precisions for all utilized ELISA kits were < 15%.

### 2.6. RNA Extraction and RT-PCR

Total RNA was extracted with TRIzol, and cDNA synthesis and RT-PCR were performed according to the manufacturer's instructions (Takara Bio, Dalian, China). Primer sequences for RT-PCR are shown in Table 1. Glyceraldehyde 3-phosphate dehydrogenase (GAPDH) was used as the housekeeping gene. The reactions were performed on a LightCycler Real-Time PCR System.

### 2.7. Western Blot

The collected cells from WRL 68 plates were washed with cold PBS, then lysed in lysis buffer (50 mM Tris/HCl, pH 7.5, 150 mM NaCl, 0.1% NP40, 1 mM EDTA) with protease inhibitors for 30 min on ice. BCA assay was used to quantitate and normalize total protein content. Proteins were then separated by 10% SDS‐PAGE gels and transferred to PVDF membranes.

Membranes were blocked with 5% BSA in TBS/0.05% Tween 20 (TBST) for 1 h at room temperature. Then, the membranes were incubated with primary overnight at 4°C, followed by the second antibody incubation for 2 h at room temperature. Each time after blocking or antibody incubation, the membranes were washed three times with TBST, each wash lasting 5 min.

The antibodies for incubation were diluted in TBST. The primary antibodies used in this study included rabbit polyclonal anti-TSHR (1 : 1000, A6781, ABclonal, China), rabbit polyclonal anti-TLR4 (1 : 1000, ab13556, Abcam, USA), rabbit polyclonal anti-p-IRAK-1 (1 : 1000, ab112042, Abcam, USA), rabbit polyclonal anti-p-NF-κB p65 (1 : 500, AF2006, Affinity, USA), rabbit polyclonal anti-Tollip (1 : 500, DF6925, Affinity, USA), mouse polyclonal anti-GLUT2 (1 : 300, bs-0351R, BIOSS, China), rabbit polyclonal anti-p-IRS-1-Tyr612 (1 : 1000, 44-816G, Thermo Fisher, USA), and mouse monoclonal anti-GAPDH (1 : 2000, ab8245, Abcam, Hong Kong). The second antibody used is peroxidase-conjugate (1 : 4000, Zhongshan Golden Bridge Biotechnology Co. Ltd., China).

### 2.8. Statistical Analyses

All analyses were performed using SPSS Statistics software, Version 23.0 (SPSS, Inc., USA). Data were expressed as mean ± SD. One-way analysis of variance (ANOVA) was used to evaluate statistical significance. When the *F* value indicated significance, Fisher's least significant difference was used to correct for multiple comparisons. *p* values < 0.05 were considered statistically significant.

## 3. Results

### 3.1. Effects of TSH/TSHR on Glucose Metabolism in Hepatocytes

**Figure 1:**
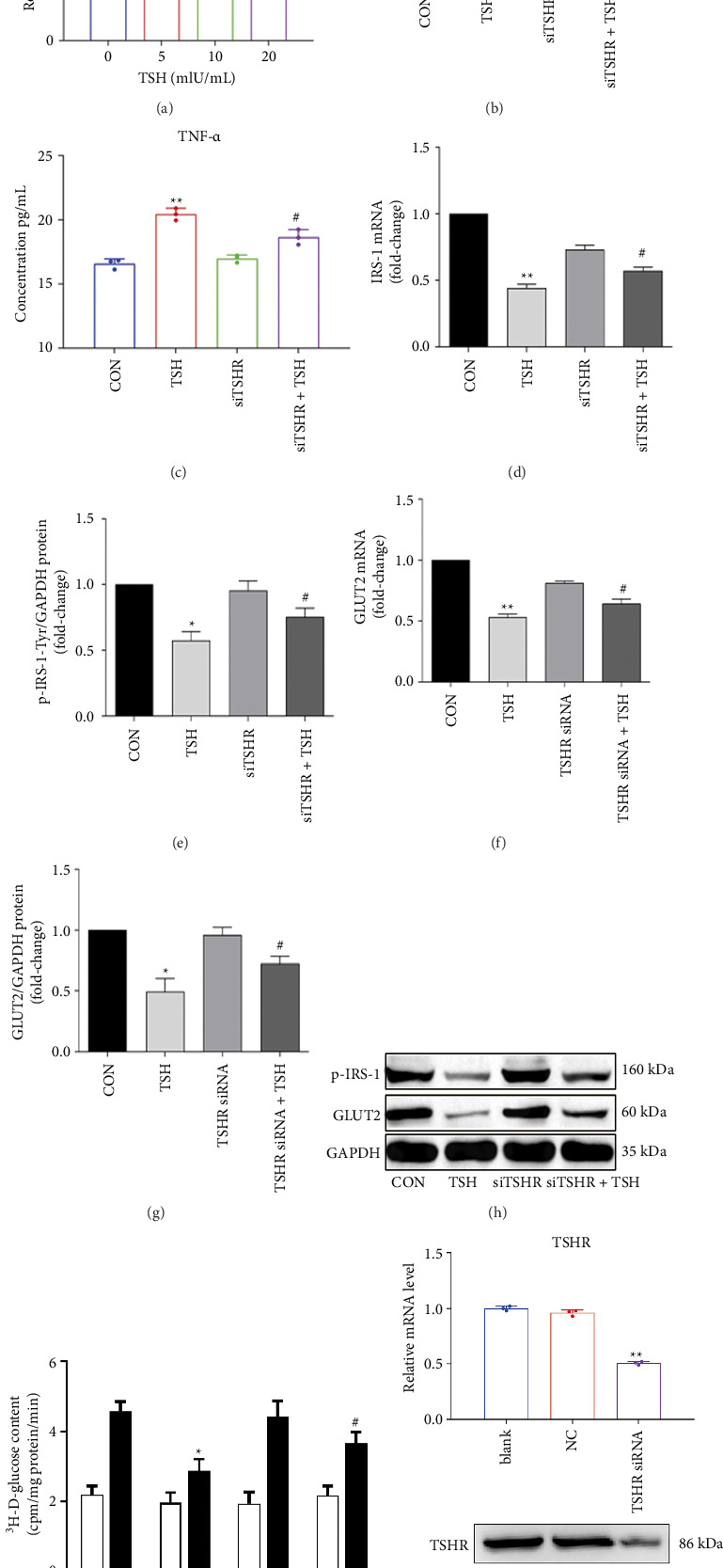
Effects of TSH/TSHR on glucose metabolism in hepatocytes. (a) TSHR mRNA levels show a gradually increasing trend when stimulated with increasing concentrations of TSH in hepatocyte. (b, c) TSH increases inflammatory cytokines IL-6 and TNF-α in the supernatant of hepatocyte. (d–g) Silencing TSHR via siRNA largely restored the IRS-1 and GLUT2 at the mRNA and protein levels. (h) Bands of proteins. (i) TSH downregulates cellular ^3^H-2-Deoxy-D-glucose uptake. (j) siRNA silencing of TSHR, the levels of TSHR mRNA and protein both significantly decreased. The mRNA levels of TSHR were measured by RT-PCR. The protein levels were measured by Western blot. The IL-6 and TNF-α concentrations were measured by ELISA. Values are expressed as mean ± SD. ^∗^*p* < 0.05 vs. control group; ^∗∗^*p* < 0.01 vs. control group. ^#^*p* < 0.05 (siTSHR + TSH) group vs. TSH group; ^##^*p* < 0.01 (siTSHR + TSH) group vs. TSH group. CON group: Hepatocyte with no-TSH (0 mIU/mL) served as the control group. The concentration of TSH was 10 mIU/mL. Control group data (mRNA and protein levels) were labeled as 1. *n* = 3 independent experimental replicates.

We first identified the optimum TSH concentration to induce TSHR expression in hepatocytes to find the best TSH treatment for TSHR expression. When stimulated with increasing TSH concentrations, we discovered that TSHR mRNA levels increased. When the TSH concentration was 10 mIU/mL or 20 mIU/mL, the TSHR expression was statistically (*p*=0.019 and = 0.0042, respectively) different from that of the control group (Figure 1(a)). For subsequent experiments, we selected a TSH dose of 10 mIU/mL (equivalent to a 1.5-fold increase in TSHR expression). The levels of cytokines IL-6 and TNF-α in culture supernatants were then examined to determine the effect of this optimal TSH administration. We found increases of approximately 5.9-fold and 1.3-fold compared to a no-TSH control, separately. Upon siRNA silencing of TSHR, the effects of subsequent TSH treatment on levels of IL-6 and TNF-α were rescued compared to the unsilenced control (Figures 1(b), 1(c), and 1(j)), implying that TSH regulates the secretion of inflammatory cytokines via TSHR. In addition, TSH (10 mIU/mL) significantly reduced the levels of IRS-1 mRNA (46.5% of control) and phosphorylated IRS-1-Tyr612 protein (57.3% of control) (Figures 1(d), 1(e), and 1(h)). For GLUT2, we found that both the mRNA (52.5% of control) and protein levels had decreased (49.5% of control). The expression of IRS-1 and GLUT2 at both the mRNA and protein levels was largely restored after TSHR was silenced (Figures 1(f), 1(g), and 1(h)).

Next, to directly investigate the impact of TSH on glucose metabolism, we measured cellular glucose uptake using ^3^H-labeled 2-deoxy-D-glucose after insulin stimulation. This glucose analog has its 2-hydroxyl group replaced by hydrogen, preventing it from undergoing further glycolysis and allowing accurate measurement. As expected, insulin-stimulated glucose uptake was significantly higher in the control group compared to untreated cells (Figure 1(i)). However, TSH stimulation (10 mIU/mL) for 12 h led to a marked reduction in glucose uptake, representing approximately 62.9% of the level observed in the unstimulated control group (*p*=0.027) (Figure 1(i)). Notably, silencing TSHR with siRNA (siTSHR) reversed this reduction, resulting in a 1.3-fold increase in glucose uptake compared to the TSH-treated group (*p*=0.034) (Figure 1(i)).

Collectively, our results suggest that TSH exerts a regulatory effect on glucose metabolism by attenuating glucose uptake via TSHR signaling in hepatocytes.

### 3.2. TLR4 Signaling is Upregulated in TSH-Stimulated Hepatocytes

**Figure 2:**
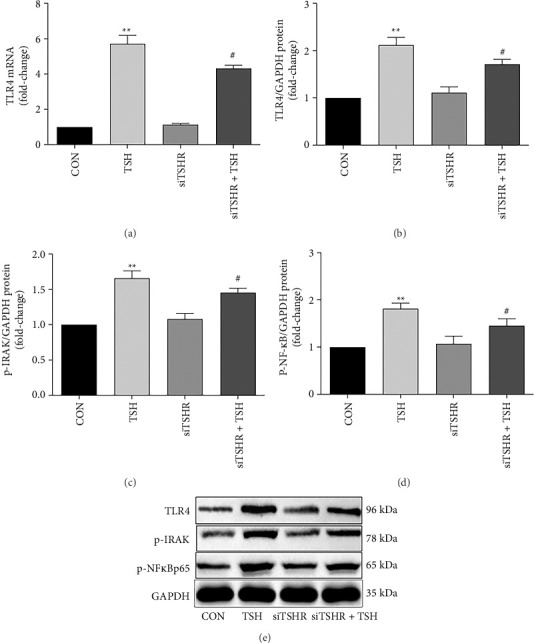
TLR4 signaling is upregulated in TSH-stimulated hepatocytes. (a–d) Silencing TSHR attenuates the TSH-mediated upregulation of the expression of TLR4 and its downstream cascades. (e) Bands of proteins. Bars represent mean ± SD. ^∗∗^*p* < 0.01: TSH group vs. CON group; ^#^*p* < 0.05 (siTSHR + TSH) group vs. TSH group. CON group: Hepatocyte with no-TSH (0 mIU/mL) served as the control group. The concentration of TSH was 10 mIU/mL. Control group data were labeled as 1. *n* = 3 independent experimental replicates.

TLR4 mRNA dramatically rose (5.7-fold) after TSH (10 mIU/mL) treatment, and so did TLR4 protein levels (2.1-fold). p-IRAK, a TLR4 regulator, was also measured, and we discovered a 1.6-fold increase in its levels, p-NF-κB protein levels increased 1.8-fold after the same treatment as well. The expression of TLR4 and its downstream partners all decreased after TSHR was silenced by siRNA in comparison with the levels in the unsilenced control treatments (TLR4 mRNA: 75.6%; TLR4: 79.1%; p-IRAK-1: 81.3%; p-NF-κB protein: 77.8%; Figure 2). Taken together, these findings indicated that TSH promotes TLR4 signaling through a TSHR-dependent mechanism.

### 3.3. TSH Downregulates Hepatocellular Glucose Metabolism via TLR4 Signaling

**Figure 3:**
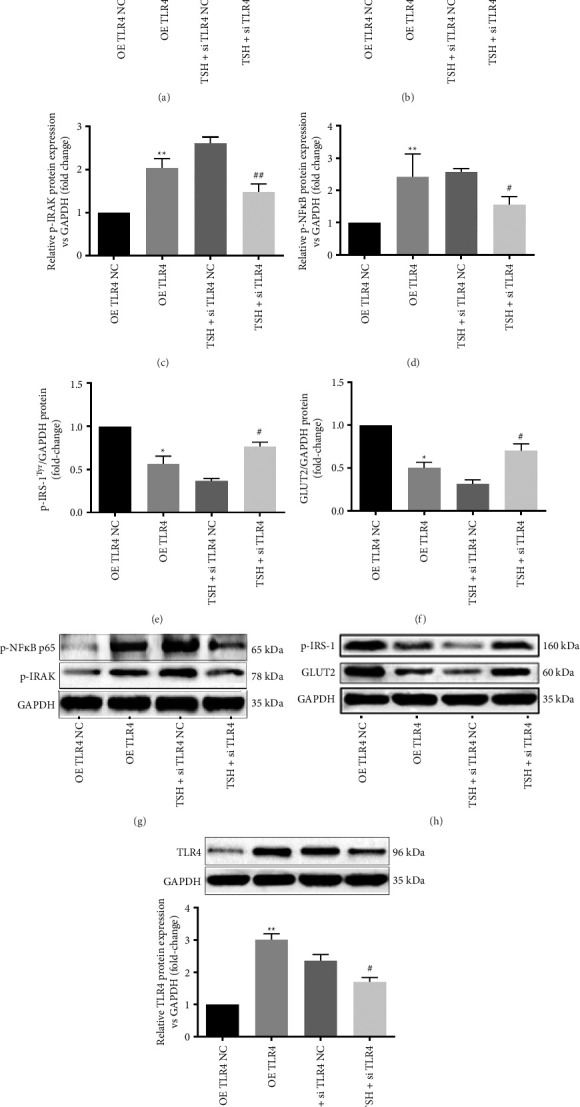
TSH downregulates hepatocellular glucose metabolism via TLR4 signaling. (a, b) Silencing of TLR4 attenuates the TSH-mediated upregulation of inflammatory cytokines IL-6 and TNF-α in the supernatant. The IL-6 and TNF-α concentrations were measured by ELISA. (c, d) Silencing of TLR4 attenuates the TSH-mediated upregulation of the expression of p-IRAK and p-NF-κB. (e, f) Silencing of TLR4 inhibits the TSH-mediated downregulation of insulin signaling. (g, h) Bands of proteins. (i) Silencing and overexpression of TLR4. Bars represent mean ± SD. ^∗^*p* < 0.05 OE TLR4 group vs. OE TLR4 NC group; ^∗∗^*p* < 0.01 OE TLR4 group vs. OE TLR4 NC group; ^#^*p* < 0.05 (TSH + Si TLR4) group vs. (TSH + Si TLR4 NC) group; ^##^*p* < 0.01 (TSH + Si TLR4) group vs. (TSH + Si TLR4 NC) group. The concentration of TSH was 10 mIU/mL. OE TLR4 NC group data were labeled as 1 (c–f). *n* = 3 independent experimental replicates.

To confirm that TSH downregulates hepatocellular glucose metabolism through TLR4 signaling, we utilized siRNA to silence TLR4 and the plasmid-based method for its overexpression. Silencing TLR4 resulted in a significant reduction in TLR4 protein levels, measuring only 37.7% of control values, while overexpression of TLR4 led to a 3.0-fold increase in TLR4 protein levels (Figure 3(i)). Following TSH stimulation, TLR4 silencing by siRNA significantly decreased the levels of TNF-α and IL-6 in the hepatocytes supernatants when stimulated with TSH (45.7% of control and 42.1% of control, respectively), while TLR4 overexpression led to higher levels of TNF-α (8.5-fold of control) and IL-6 (15.8-fold of control). Each of the differences was statistically significant (Figures 3(a), 3(b), and 3(i)), indicating that TSH mediated the inflammatory cytokines via TLR4.

Furthermore, when TLR4 was silenced, TSH stimulation led to significant reductions of p-IRAK (53.9% of the control) and p-NF-κB (64.2% of the control) (Figures 3(c), 3(d), and 3(g)). Additionally, TLR4 overexpression resulted in a significant reduction in p-IRS-1-Tyr^612^ protein levels (56.3% of the control) and GLUT2 protein levels (50.3% of the control). Conversely, upon silencing of TLR4 overexpression, the levels of p-IRS-1-Tyr^612^ and GLUT2 proteins both increased significantly (2.1-fold and 2.3-fold over control; Figures 3(e), 3(f), and 3(h)).

In summary, these results confirm that TLR4 involved in is hepatocytes' response to TSH stimulation, acting as a downstream mediator that activates inflammatory cytokine production and glucose metabolism genes in hepatocytes.

Tollip attenuates the TSH-mediated upregulation of TLR4 signaling and thus upregulates glucose metabolism in hepatocytes.

**Figure 4:**
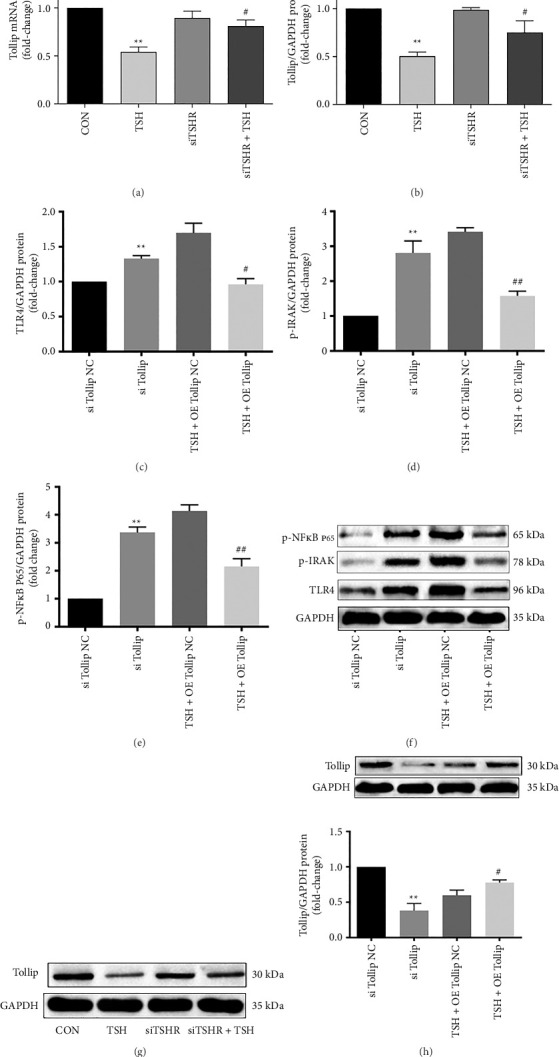
Tollip attenuates the TSH-mediated upregulation of TLR4 signaling in hepatocytes. (a, b) Silencing TSHR via siRNA increased the expression of Tollip. (c–e) Overexpression of Tollip attenuates the TSH-mediated upregulation of the expression of TLR4 and its downstream cascades. (f, g) Bands of proteins. (h) The levels of Tollip protein of silencing and overexpression. Bars represent mean ± SD. ^∗∗^*p* < 0.01 si Tollip group vs. si Tollip NC group and TSH group vs. CON group. ^#^*p* < 0.05 (TSH + OE Tollip) group vs. (TSH + OE Tollip NC) group and (SiTSHR + TSH) group vs. TSH group; ^##^*p* < 0.01 (TSH + OE Tollip) group vs. (TSH + OE Tollip NC) group. The concentration of TSH was 10 mIU/mL. Control group data were labeled as 1. *n* = 3 independent experimental replicates.

We examined how TSH treatment affected the mRNA level of Tollip and discovered a significant decrease (51.2% of the control group). In contrast, Tollip's mRNA level increased 1.7-fold when TSHR was silenced (Figure 4(a)). Meanwhile, Tollip protein decreased to 49.8% after TSH treatment and increased 1.6-fold after TSHR silencing (Figures 4(b) and 4(g)).

Next, we investigated the role of Tollip in TLR4-mediated TSH responses by manipulating its levels. Similar to our approach with TLR4, we employed siRNA silencing, which reduced Tollip protein levels to 38.5% of the control, and plasmid-based overexpression, which led to a 1.5-fold increase in Tollip protein levels (Figure 4(i)). Upon siRNA silencing of Tollip, TLR4 protein levels increased significantly (1.9-fold over the control) as did protein levels of p-IRAK (2.8-fold over control) and p-NF-κB (3.3-fold over control). TLR4 protein decreased significantly (56.9% of the control) in response to Tollip overexpression, as did those of downstream partners p-IRAK (45.3% of control) and p-NF-κB (52.2% of control; Figures 4(c), 4(d), 4(e), 4(f), and 4(h)). These results indicated that Tollip attenuates the TSH-mediated upregulation of TLR4 signaling in hepatocytes.

**Figure 5:**
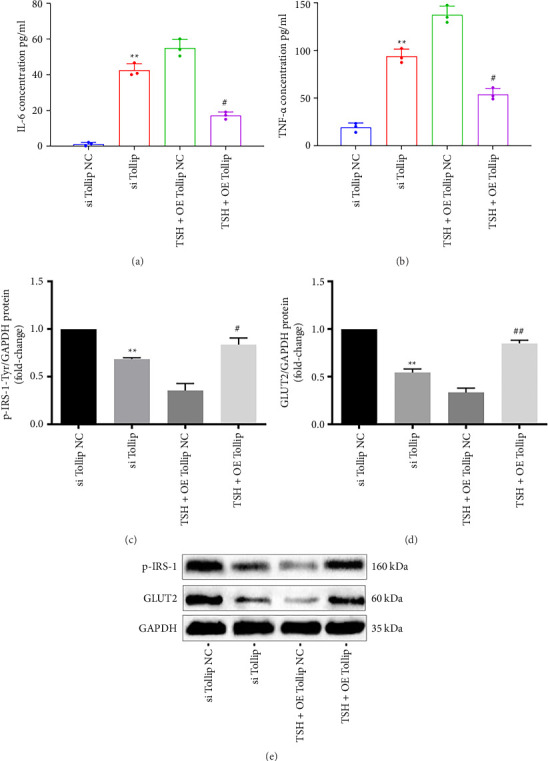
Tollip attenuates the TSH-mediated downregulation of glucose metabolism in hepatocytes. (a, b) The IL-6 and TNF-α concentrations were measured by ELISA. (c, d) Overexpression of Tollip inhibits the TSH-mediated downregulation of insulin signaling. (e) Bands of proteins. Bars represent mean ± SD. ^∗∗^*p* < 0.01 si Tollip group vs. si Tollip NC group. ^#^*p* < 0.05 (TSH + OE Tollip) group vs. (TSH + OE Tollip NC) group; ^##^*p* < 0.01 (TSH + OE Tollip) group vs. (TSH + OE Tollip NC) group. The concentration of TSH was 10 mIU/mL. Control group data were labeled as 1. *n* = 3 independent experimental replicates.

TNF-α and IL-6 protein levels in hepatocyte culture supernatants increased significantly (4.9-fold and 37.3-fold, respectively) after Tollip was silenced, whereas Tollip overexpression resulted in lower levels of TNF-α (39.3% of control) and IL-6 protein (31.2% of control; Figures 5(a) and 5(b)), implying that Tollip attenuates the TSH-mediated upregulation of inflammatory cytokines. Both p-IRS-1-Tyr^612^ and GLUT2 protein levels were reduced significantly (68.7% and 54.1% of control) after Tollip was silenced. Upon Tollip overexpression, the levels of p-IRS-1-Tyr^612^ protein were increased (2.4-fold over control), as were GLUT2 (2.6-fold over control) (Figures 5(c), 5(d), and 5(e)). These results indicated that Tollip attenuates the TSH-mediated downregulation of glucose metabolism in hepatocytes.

## 4. Discussion

Our study shows that TSH blocks TSHR-mediated glucose metabolism in hepatocytes via TLR4/Tollip pathway. We find that TSH binding induces IRAK phosphorylation, thus activating the TLR4/NF-κB pathway and promoting the secretion of the proinflammatory cytokines, TNF-α and IL-6, that lead to chronic inflammation. TSH also inhibited the insulin signal transduction pathway via suppression of GLUT2 and p-IRS-1-Tyr levels. Tollip, a negative regulator of TLR4 signaling, attenuated the TSH-mediated induction of TLR4 signaling, thereby rectify dysfunction of glucose metabolism in hepatocytes [24–26]. Taken together, these results elucidate the underlying mechanism of TSH in regulating hepatic TLR4 signaling and glucose metabolism.

Within the thyroid gland, TSH binds to the G-protein-coupled TSHR to induce intracellular signaling, and this receptor has also been identified in the liver, adipocytes, ovaries, ocular muscles, and immune cells, though its role in these extra-thyroid tissues has been unclear [27]. Signaling from TSH/TSHR may regulate thermogenesis, adipogenesis, and lipolysis/lipogenesis balance in adipocytes [28, 29], though conflicting finds leave unclear the relationship between TSHR expression and body weight [30, 31]. Research has found that when adipocytes are stimulated with different concentrations of TSH, they release cAMP and glycerol in proportion to the TSH concentration, suggesting that TSH may act through a cAMP signaling pathway in adipocytes [32]. In our study, the levels of TSHR mRNA in hepatocytes increased gradually when treated with increasing concentrations of TSH. TSH stimulation can upregulate the activity of TLR4 signaling. When the expression of TSHR is suppressed, TSH activity is blocked. The results indicate that TSH regulates the TLR4 inflammatory signaling pathway of hepatocytes via a concentration-dependent receptor.

Signaling through the TLR4 pathway is known to play an essential role in multiple liver diseases [33, 34]. TLRs recognize molecular patterns associated with pathogens and activate downstream signaling cascades (e.g., IKK, NF-κB) via pathway-specific adaptor proteins, thereby inducing the production of enzymes, proinflammatory cytokines, interferons, and chemokines [34]. The TLR4 pathways are regulated by transmembrane proteins, soluble receptors, and intracellular inhibitors to balance the effects of pathogen antigen recognition [19, 35]. Here, we found that TSH activates TLR4/NF-κB signaling to promote the secretion of proinflammatory cytokines like TNF-α and IL-6 in hepatocytes, though the exact mechanism remains to be determined.

**Figure 6:**
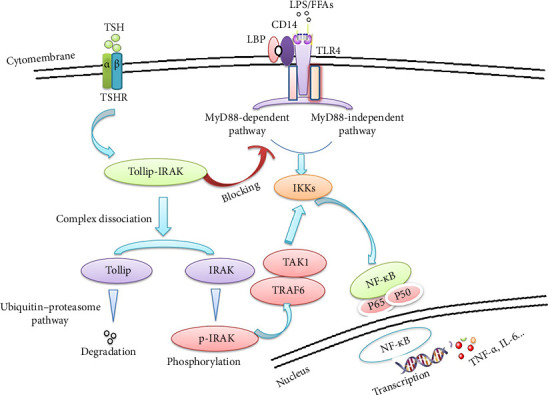
A diagram showing molecular mechanism for Tollip attenuating the TSH-mediated upregulation of TLR4 signaling. TSH facilitates the signaling of TLR4 through a mechanism that is dependent on the TSHR. Tollip, which serves as an inhibitor of the TLR signaling pathway, typically forms a complex with IRAK-1 under normal conditions. This interaction inhibits the MyD88-dependent pathway within the TLR signaling cascade by preventing the phosphorylation of IRAK-1. Upon TSH stimulation, the expression of TSHR is induced, leading to the targeted degradation of Tollip through the process of ubiquitination. This degradation results in the dissociation of Tollip from IRAK-1. This dissociation allows the phosphorylation of IRAK, thereby activating the TLR signals. The activation subsequently promotes the translocation of NF-κB into the nucleus and finally induces the transcription of proinflammatory cytokines like TNF-α and IL-6, thereby increasing the inflammatory state of tissue. The increased expression of IL-6 and TNF-α can potentially impact insulin signaling.

Tollip bridges autophagosomes to cargo via a ubiquitin-binding domain and a post-translationally modified LC3-interacting region [18, 36]. Tollip protein has many effects. It alleviates intestinal mucosal inflammation in mice by orchestrating macrophage polarization via TLR pathway attenuation [37], promotes hepatocellular carcinoma progression via the PI3K/AKT pathway [38], attenuates the hypertrophic response of neonatal cardiomyocytes through negative regulation of the MyD88-dependent NF-κB pathway [39], and acts as a novel therapeutic target for hepatic ischemia–reperfusion injury in mice [25]. However, the effect of Tollip on hepatocytes in SCH has been unclear. In the present study, we found an attenuating effect on TLR4, which might be considered as the evidence to prove the potential effect to diminish in SCH. We have demonstrated that TSH-promoted inflammation and insulin resistance are associated with Tollip protein through its regulation of TLR4/NF-κB signaling (Figure 6), thereby suggesting an avenue of therapeutic intervention for adjusting the dysfunction of glucose metabolism.

The mechanism of dysfunction of glucose metabolism is very complex, with NLR (NOD-like receptor signaling pathway), TLR, AKT, and other pathways all implicated in insulin signaling [40–42]. Though we identified a TSH-to-TLR4 pathway for hepatocyte insulin signaling, we have yet to determine whether TSH may also regulate insulin signal via other signaling pathways like the NLR-regulated pathway in SCH-related dysfunction of glucose metabolism. These studies illuminate new connections between TSH, glucose metabolism, and immune-related inflammation, thereby revealing a pathophysiological mechanism of inflammation and dysfunction of glucose metabolism and suggesting a therapeutic route.

This study has several limitations. First, while our findings highlight a TSH–TLR4–Tollip axis in regulating hepatocyte glucose metabolism, the regulatory effects of TSH on insulin signaling through other pathways (e.g., NLR or AKT signaling) were not fully addressed. Second, although we observed that TSH promotes the secretion of proinflammatory cytokines such as TNF-α and IL-6, the precise molecular mechanism underlying TSH activation of these cytokines remains to be determined. Future studies are needed to explore these pathways in greater detail and to establish the broader network by which TSH contributes to SCH-related dysfunction of glucose metabolism. Our previous work demonstrated that SCH aggravates TLR4 signaling and disrupts glucose metabolism in rat liver tissue [24]. The present study in human hepatocytes provides proof-of-concept in a human cellular model; however, in vivo validation using human liver samples or patient cohorts will be essential to establish the clinical relevance of TSH-mediated regulation of glucose metabolism.

In conclusion, our study identifies TSH as a key regulator of hepatocyte glucose metabolism through the TLR4/Tollip signaling pathway, linking inflammation with insulin resistance in the context of SCH. These findings provide new mechanistic insight into the role of TSH in hepatic dysfunction and highlight potential therapeutic targets for improving glucose metabolism in SCH.

## Figures and Tables

**Table 1:** Primer design.

Genes	Primer sequences
TSHR	Forward: 5ʹ-CTTGGCATTTTCAACACTGG-3ʹ
	Reverse: 5ʹ-AAGGTTTCATTGCATAGTCCC-3ʹ

Tollip	Forward: 5ʹ-GACTGAACATCACGGTGGTAC-3ʹ
	Reverse: 5ʹ-GAGATAGAAAGAGTCCACGCC-3ʹ

TLR4	Forward: 5ʹ-TTTAGACCTGTCCCTGAACCC-3ʹ
	Reverse: 5ʹ-CTAAACCAGCCAGACCTTGAA-3ʹ

IRS-1	Forward: 5ʹ-GTCCTCCTCGCTCTTGTCC-3ʹ
	Reverse: 5ʹ-CTTAGGAGACTTGGGGGAGC-3ʹ

GLUT2	Forward: 5ʹ-CAGCTGCTCAACTAATCACCA-3ʹ
	Reverse: 5ʹ-CCAATTTTGAAAACCCCATC-3ʹ

GAPDH	Forward: 5ʹ-GCCTTCCGTGTCCCCACTGC-3ʹ
	Reverse: 5ʹ-GGCTGGTGGTCCAGGGGTCT-3ʹ

Abbreviations: GAPDH = glyceraldehyde-3-phosphate dehydrogenase, GLUT2 = glucose transporter 2, IRS-1 = insulin receptor substrate-1, TLR4 = Toll-like receptor 4, Tollip = Toll-interacting protein, TSHR = thyroid-stimulating hormone.

## Data Availability

The datasets used and/or analyzed during the current study are available from the corresponding author on reasonable request.

## Nomenclature

CD14: Cluster of differentiation 14
GLUT2: Glucose transporter 2
IL-6: Interleukin-6
IKKs: Inhibitory nuclear factor-kappa-B kinase
MyD88: Myeloid differentiation factor 88
p-IRAK: Phosphorylated interleukin receptor-related kinase
p-IRS-1-Tyr: Insulin receptor substrate-1 tyrosine phosphorylation
p-NF-κB: Phosphorylated nuclear factor-kappa-B
SCH: Subclinical hypothyroidism
TAK1: Transforming growth factor beta-activated kinase 1
TLR4: Toll-like receptor 4
Tollip: Toll-interacting protein
TNF-α: Tumor necrosis factor-α
TRAF6: Tumor necrosis factor receptor-associated factor-6
TSH: Thyroid-stimulating hormone
